# Managing Advanced HIV Disease in a Public Health Approach

**DOI:** 10.1093/cid/cix1139

**Published:** 2018-03-04

**Authors:** Nathan Ford, Graeme Meintjes, Alexandra Calmy, Helen Bygrave, Chantal Migone, Marco Vitoria, Martina Penazzato, Lara Vojnov, Meg Doherty, Guideline Development Group for Managing Advanced HIV Disease and Rapid Initiation of Antiretroviral Therapy, Patricia Asero, Rosa Bologna, Mohamed Chakroun, Lucia Chambal, Tom Chiller, Francesca Conradie, Serge Eholie, Lisa Frigati, Diana Gibb, Eric Goemaere, Nelesh Govender, Alison Grant, Nagalingeswaran Kumarasamy, David Lalloo, Thuy Le, Emilio Letang, Dorothy Mbori-Ngacha, Sayoki Mfinanga, Mathieu Nacher, Muhayimpundu Ribakare, Nandi Siegfried, Kenly Sikwese, Nini Tun, Jose E Vidal

**Affiliations:** 1 HIV Department, World Health Organization, Geneva, Switzerland; 2 Wellcome Trust Centre for Infectious Diseases Research in Africa, Institute of Infectious Disease and Molecular Medicine; 3 Department of Medicine, Faculty of Health Sciences, University of Cape Town, South Africa; 4 Division of Infectious Diseases, HIV Unit, Department of Internal Medicine Specialties, Geneva University Hospitals and Faculty of Medicine, Switzerland; 5 Médecins Sans Frontières, Southern Africa Medical Unit, Cape Town, South Africa

**Keywords:** advanced HIV disease, cryptococcal meningitis, tuberculosis

## Abstract

In 2017, the World Health Organization (WHO) published guidelines for the management of advanced human immunodeficiency virus (HIV) disease within a public health approach. Recent data suggest that more than a third of people starting antiretroviral therapy (ART) do so with advanced HIV disease, and an increasing number of patients re-present to care at an advanced stage of HIV disease following a period of disengagement from care. These guidelines recommend a standardized package of care for adults, adolescents, and children, based on the leading causes of morbidity and mortality: tuberculosis, severe bacterial infections, cryptococcal meningitis, toxoplasmosis, and *Pneumocystis jirovecii* pneumonia. A package of targeted interventions to reduce mortality and morbidity was recommended, based on results of 2 recent randomized trials that both showed a mortality reduction associated with delivery of a simplified intervention package. Taking these results and existing recommendations into consideration, WHO recommends that a package of care be offered to those presenting with advanced HIV disease; depending on age and CD4 cell count, the package may include opportunistic infection screening and prophylaxis, including fluconazole preemptive therapy for those who are cryptococcal antigen positive and without evidence of meningitis. Rapid ART initiation and intensified adherence interventions should also be proposed to everyone presenting with advanced HIV disease.

The annual number of people dying from AIDS-related causes has declined by 48% since 2003, with 1 million AIDS-related deaths reported in 2016 [1]. This decline is largely the result of expanded access to human immunodeficiency virus (HIV) testing and antiretroviral therapy (ART) and an evolution toward treating people earlier in the course of HIV infection.[2]

Notwithstanding this progress, the decline in AIDS-related deaths appears to have plateaued in recent years. This is largely due to the persistent challenge of advanced HIV disease. People presenting with advanced HIV disease—defined by the World Health Organization (WHO) as having a CD4 cell count <200 cells/µL or stage III or IV disease; all children <5 years with HIV are considered to have advanced HIV disease [3]—are at high risk of opportunistic disease and death, even after starting ART, with the risk increasing with decreasing CD4 cell count [4, 5]. In a recent study from Kenya, Malawi, Uganda, and Zimbabwe, almost half of the people with CD4 count <100 cells/µL were classified as having WHO clinical stage 1 or 2 disease. Hence, identifying people with advanced HIV disease who are eligible for elements of a package of care requires CD4 cell count testing [6].

Recent data from South Africa show that more than a third of people starting ART do so with advanced HIV disease [7]—a trend also observed in many other countries in low-, middle-, and high-income settings [8]. In addition, there is a growing realization that an increasing proportion of people with advanced HIV disease is represented by patients who had previously engaged with the health system and started ART, and subsequently disengaged from care. Finally, there are those who experience ART failure without disengagement [9, 10].

In 2017 WHO convened an expert group to review the evidence and formulate recommendations for the management of advanced HIV disease. The objective of these guidelines is to define a package of evidence-informed interventions that can be provided to people with advanced HIV disease as part of a public health approach to managing HIV infection. While the focus of the WHO guidelines is on countries with limited resources and the greatest burden of disease, the recommendations are intended to apply to all settings where advanced HIV disease continues to be an important challenge. The elements of the core package are summarized in Table 1.

**Table 1.** Components of the Package of Care for People With Advanced Human Immunodeficiency Virus Disease

Intervention Area	Intervention	CD4 Cell Count	Adults and Adolescents	Children
Screening and diagnosis	Sputum Xpert MTB/RIF assay as first test for TB diagnosis in symptomatic patients	Any	Yes	Yes
	Urine LF-LAM for TB diagnosis in patients with symptoms and signs of TB	≤100 cells/µL or at any CD4 cell count value if seriously ill	Yes	Yes^a^
	CrAg screening	<100 cells/µL^b^	Yes	No
Prophylaxis and preemptive treatment	Cotrimoxazole prophylaxis^c^	≤350 cells/µL or WHO clinical stage 3 or 4 event. Any CD4 cell count value in settings with high prevalence of malaria and/or severe bacterial infections	Yes	Yes^d^
	TB preventive treatment^b^	Any	Yes	Yes^e^
	Fluconazole preemptive therapy for CrAg- positive patients without evidence of meningitis	<100 cells/µL	Yes	Not applicable (screening not advised)
ART initiation	Rapid ART initiation	Any	Yes	Yes
	Defer ART initiation if clinical signs and symptoms are suggestive of TB or cryptococcal meningitis	Any	Yes	Yes
Adapted adherence support	Tailored counseling to ensure optimal adherence to advance disease care package, including home visits if feasible	< 200 cells/µL	Yes	Yes

Abbreviations: ART, antiretroviral therapy; CrAg, cryptococcal antigen; LF-LAM, lateral flow lipoarabinomannan assay; TB, tuberculosis; WHO, World Health Organization.

^a^Limited data available for children.

^b^This threshold was revised upward in 2018 to include people with CD4 <200 cells/µL.

^c^Cotrimoxazole, isoniazid, and pyridoxine are available as a fixed-dose combination tablet.

^d^Priority should be given to all children <5 years old regardless of CD4 cell count or clinical stage, and those with severe or advanced human immunodeficiency virus clinical disease (WHO clinical stage 3 or 4 event and /or those with CD4 ≤350 cells/µL).

^e^For children <12 months of age, only those with a history of TB contact should receive TB preventive treatment if the evaluation shows no active TB disease.

## PRIORITIES FOR THE MANAGEMENT OF ADVANCED HIV DISEASE

Leading causes of mortality among adults with advanced HIV disease globally include tuberculosis (TB), severe bacterial infections, cryptococcal meningitis, toxoplasmosis, and *Pneumocystis jirovecii* pneumonia (PCP) [5, 11, 12].

Two randomized clinical trials have been carried out to assess the effectiveness of a package of interventions aimed at reducing mortality and morbidity among people with advanced HIV disease [6, 13].

The first trial (REMSTART), conducted in the United Republic of Tanzania and Zambia, randomized 1999 ART-naive adults living with HIV with CD4 count <200 cells/µL to receive enhanced clinic-based care with a point-of-care serum cryptococcal antigen (CrAg) screening and preemptive antifungal treatment for those who tested CrAg positive and additional community support compared with standard care. In this trial, the combination of 4 short home or community visits by lay workers combined with CrAg screening followed by treatment for cryptococcal antigenemia led to a 28% (95% confidence interval [CI], 10%–43%) reduction in mortality among people presenting with advanced HIV disease (mortality was 13% in the intervention group vs 18% in the group receiving standard care; *P* = .004), and a decreased rate of hospitalization. A tendency toward improved ART adherence in the intervention group compared with the standard-of-care group was reported at 6 months, but no difference was noted by 12 months on ART [13]. No formal economic evaluation has yet been conducted, but a microcosting study found that the enhanced package represented a 55% increase in the mean cost of care over the first 3 months, falling to 25% over 1 year of follow-up [14].

The second trial (REALITY) enrolled 1805 participants (mainly adults) living with HIV with CD4 counts <100 cells/µL (median CD4 count was 36 cells/µL and half were classified as WHO clinical stage 1 or 2) from Kenya, Malawi, Uganda, and Zimbabwe. Trial participants were randomized to the standard of care (cotrimoxazole) according to national guidelines or an enhanced prophylaxis package (12 weeks of fluconazole [100 mg once daily], 12 weeks of a fixed-dose combination of cotrimoxazole [800 + 160 mg] + isoniazid [300 mg] + pyridoxine [25 mg] as a scored once-daily tablet, 5 days of 500 mg of azithromycin once daily, and a single dose of 400 mg of albendazole). The doses were halved for children 5–12 years old (except for albendazole). All drugs were started simultaneously in the absence of any blood tests, and ART was offered on the same day as the prophylaxis package. Isoniazid + pyridoxine use beyond 12 weeks followed national guidelines. Participants already receiving or needing antimicrobial treatment or prophylaxis pragmatically received it outside the randomized design, and received other prophylaxis according to randomization. Patients who had started the enhanced-prophylaxis package at the time of ART initiation had a 27% lower rate of death than those who received standard prophylaxis with cotrimoxazole alone (8.9% vs 12.2%; hazard ratio, 0.73 [95% CI, .55–.98]; *P* = .03). Incidence of TB was significantly lower with enhanced prophylaxis than with standard prophylaxis (7.1% vs 10.2%. respectively), as was the incidence of cryptococcal disease (1.0% vs 2.6%), candidiasis (1.1% vs 2.6%), and new hospitalization (17.0% vs 20.7%) [6]. In the main trial report, it was concluded that the REALITY package would fall within cost-effectiveness thresholds for low-income countries [6]. More extensive costing analyses are under way.

Based on the results of these 2 trials, the WHO Guideline Development Group (GDG) for advanced HIV disease recommended providing a package of targeted interventions to reduce mortality and morbidity among people presenting with advanced HIV disease.

During the GDG meeting, concern was expressed about the available evidence to support the specific interventions assessed in the trials to be considered for the package. Although severe bacterial infections are recognized as a common and frequently overlooked cause of HIV-associated morbidity and mortality, the GDG did not consider that there is currently enough evidence to recommend including an additional broad-spectrum antibiotic (azithromycin in addition to cotrimoxazole) within the package and that the unclear benefits of this specific component of the REALITY package (mortality reduction could not clearly be attributed to a decline in bacterial infections) do not outweigh concerns about the potential for antimicrobial resistance development; financial and supply chain considerations were also taken into consideration. With respect to use of primary fluconazole prophylaxis for preventing cryptococcal disease in people presenting with advanced HIV disease, it was recognized that in settings where cryptococcal screening tests are not available or results will be delayed, fluconazole prophylaxis may offer programmatic benefits after ruling out pregnancy, with a proven reduction in mortality and the incidence of cryptococcal disease.

Taking this into consideration, WHO strongly recommends that a package of care be offered to those presenting with advanced HIV disease; depending on age and CD4 cell count, the package may include opportunistic infection screening and prophylaxis, including fluconazole preemptive therapy for those who are CrAg positive and without evidence of meningitis, TB symptom screen, diagnostic workup, treatment when appropriate, and TB preventive therapy in those without TB symptoms. Finally, rapid ART initiation and intensified adherence interventions should also be proposed to everyone presenting with advanced HIV disease [3] (Table 1). 

## WHO CAN OFFER THE PACKAGE OF CARE FOR ADVANCED HIV DISEASE?

Task sharing may be considered both for performing the point-of-care diagnostics within the package and for clinical management. A good practice statement within the 2016 WHO consolidated antiretroviral guidelines states that trained and supervised nonlaboratory staff, including lay people, can undertake blood finger-prick for testing and sample collection [15]. Lay workers have successfully delivered CrAg testing [16] and point-of-care CD4 cell count [17], and this approach should be considered as a way to improve access to essential diagnostics for the management of advanced HIV disease at peripheral sites. Task sharing to nurses and other midlevel health workers for the clinical management of people with advanced HIV should be supported with training and mentorship. Initial screening procedures, treatment of TB and some opportunistic infections, and prophylaxis may all be provided within a well-supported, nurse-led program. However, clear referral criteria and care pathways must be in place to ensure appropriate investigation and higher-level clinical management when required. Where referral is not feasible, lower-level health workers should consider presumptive treatment based on the clinical presentation, ideally after consulting with an experienced clinician.

## INTENSITY OF FOLLOW-UP FOR PEOPLE WITH ADVANCED HIV DISEASE

People with advanced HIV disease require closer follow-up during the initial period of receiving ART to monitor the response to ART and to identify signs and symptoms of possible immune reconstitution inflammatory syndrome. During the REMSTART study, weekly home visits were provided during the first month on ART [13]. Even with close initial follow-up in the REALITY trial, many people died at home, with most deaths occurring very soon after ART initiation. People discharged after hospitalization for advanced HIV disease may also require more intensive follow-up. The feasibility of more frequent visits is context specific and may also depend on the person’s ability to travel to the clinical site. People missing appointments should also be rapidly traced by phone or through home visits. Where face-to-face contact is not feasible, distance contact (through, eg, telephone consultation, mobile health, text messaging) or visits by a community health worker or home-based caregiver should be considered, with consent of the client. For hospitalized patients, programs should provide measures to improve linkage and follow-up after discharge such as outpatient primary care clinic visits and home visits by community health workers to reduce the risks of loss to follow-up and mortality after discharge.

## CONSIDERATIONS FOR SPECIFIC POPULATIONS

The advanced disease package is based on evidence from trials conducted in ART-naive, mainly adult patients in southern Africa.

For children, the major causes of mortality and morbidity among those with advanced HIV disease are TB, severe bacterial infections, and PCP but, in contrast with adults, cryptococcal disease is relatively rare. For this reason, extrapolation from adult studies is not appropriate and further research is needed to determine the components of the package of care for young children and optimal administration and delivery in this age group [18].

For ART-experienced patients with advanced HIV who have disengaged from care and then return or are failing their current regimen, the key questions relate to choice of ART regimen and timing of any switch. Restarting ART rapidly is of utmost importance, except in the presence of cryptococcal or tuberculosis meningitis. If ART interruption was complete (due, eg, to some travel duties or migration), restarting the first-line ART is possible. If partial ART exposure is suspected or the patient is severely ill, an expedited switch to a new regimen when needed should be facilitated by reducing the time between the first and second viral load test (1–3 months) and by paying increased attention to ensuring rapid turnaround and action on the results. Where rapid viral load testing is not available, the decision to switch should be assessed according to the individual clinical presentation and adherence history of the patient [19].

Finally, there are important regional differences with respect to coinfections. Fungal infections, notably histoplasmosis and talaromycosis, are associated with advanced HIV disease in specific geographical areas. In some high-income countries, advanced liver disease secondary to hepatitis B and C is among the leading infectious causes of mortality. The advanced HIV disease package should therefore take into account the need for adapting interventions according to local epidemiology.

## RESEARCH PRIORITIES

The WHO guideline development process offers an opportunity to identify priority areas for future research [20]. These are summarized in Table 2. Research priorities related to diagnostics include the need for a simplified tool to perform CD4 cell count testing to ensure that people with advanced immune disease are identified. Ongoing research is being undertaken, including using a semiquantitative CD4 cell count lateral flow assay for which operational research will be required to evaluate performance and feasibility under field conditions. Further research is also needed to develop simplified point-of-care diagnostics, including for TB and nontuberculous mycobacterial diseases, severe bacterial infections, PCP, toxoplasmosis, cytomegalovirus disease, and other opportunistic infections specific to geographical regions, such as histoplasmosis and talaromycosis. Enhanced TB screening with Xpert MTB/RIF assay, regardless of symptoms, was provided in both groups in the REMSTART study [13] and its impact could therefore not be assessed. Further studies are needed to assess improved strategies for TB screening and clarify the role of presumptive TB treatment.

**Table 2.** Research Priorities

Intervention Area	Priority
General	• Effectiveness of the package of care for ART-experienced patients
	• Defining the optimal package of care for children
Diagnosis	• Simplified tool to perform CD4 cell count testing
	• Point-of-care diagnostics for TB and nontuberculous mycobacteria, severe bacterial infections, *Pneumocystis jirovecii* pneumonia, toxoplasmosis, cytomegalovirus disease
	• Strategies to improve TB screening
	• Approaches to improve accessibility of brain imaging
Prophylaxis	• Optimal package of prophylactic interventions for people who have not yet started ART and those returning to care after interruption
	• Role of routine fluconazole prophylaxis vs CrAg screening and preemptive treatment of CrAg-positive patients in operational settings
	• Role of additional antibiotic prophylaxis for severe bacterial infections
Treatment	• Monitoring and switching strategies for patients representing and restating ART after a period of disengagement from care
	• Simple tests to assess ART adherence

Abbreviations: ART, antiretroviral therapy; CrAg, cryptococcal antigen; TB, tuberculosis.

Other research priorities include defining the optimal package of prophylactic interventions for people with advanced HIV disease, the optimal preemptive treatment strategy for people identified as CrAg positive at screening, and benefits and risks of routine use of broad-spectrum antibiotic therapy (in addition to cotrimoxazole) within a public health approach, and specifically, the independent effect of short-course azithromycin on mortality. The GDG also highlighted programmatic assessment of the intensity of follow-up required and adherence strategies for people with advanced HIV disease as important areas for implementation research.

To date, trials have only examined the benefits of an intervention package among those presenting for ART care without previous ART exposure. The optimal management approach for people re-presenting for care with advanced HIV disease after treatment interruption warrants further investigation. Data to support the effectiveness and safety of intervention packages in routine care settings would also be of value. In addition, as these trials have not investigated the benefit of an intervention package for infants and children <5 years of age, specific components and optimal delivery in this population warrant further research. Finally, region-specific packages of care should be defined and their effectiveness assessed.

## CONCLUSIONS

Continued progress in improving access to HIV diagnosis and treatment remains at the core of the global response toward achieving the target of ending AIDS as a public health threat and providing universal health coverage. Nevertheless, HIV infection remains a leading cause of illness and death in many countries, particularly in sub-Saharan Africa, and evidence to date suggests that responding to advanced HIV disease will remain an important public health priority for several years to come. National HIV programs are encouraged to implement the package of interventions recommended by WHO, and WHO will update this package according to new evidence and implementation experience.

## Notes

***Acknowledgments.*** WHO is grateful to the many peer reviewers who provided valuable comments in support of the development of these guidelines. The following people participated in the WHO: Guideline Development Group for managing advanced HIV disease and rapid initiation of antiretroviral therapy: Eduardo Arathoon (Asociacion de Salud Integral, Guatemala), Patricia Asero (International Community of Women Living with HIV, Kenya), Rosa Bologna (Hospital de Pediatría Prof Dr Juan P. Garrahan, Argentina), Mohamed Chakroun (Fattouma Bourguiba Teaching Hospital, Tunisia), Lucia Chambal (Ministry of Health, Mozambique), Tom Chiller (Mycotic Disease Branch, US Centers for Disease Control and Prevention [CDC]), Francesca Conradie (University of the Witwatersrand, South Africa), Serge Eholie (Centre Hospitalier Universitaire de Treichville, Côte d’Ivoire), Lisa Frigati (Tygerberg Hospital and Stellenbosch University, South Africa), Diana Gibb (Medical Research Council, United Kingdom), Eric Goemaere (Médecins Sans Frontières, South Africa), Nelesh Govender (University of the Witwatersrand and National Institute for Communicable Diseases, South Africa), Alison Grant (London School of Hygiene and Tropical Medicine, United Kingdom), Nagalingeswaran Kumarasamy (YRGCARE, India), David Lalloo (Liverpool School of Tropical Medicine, United Kingdom), Thuy Le (Oxford University Clinical Research Unit, Viet Nam), Emilio Letang (Barcelona Institute for Global Health and Ifakara Health Institute, Spain), Dorothy Mbori-Ngacha (UNICEF, Kenya), Sayoki Mfinanga (Muhimbili Medical Research Centre, National Institute for Medical Research, United Republic of Tanzania), Mathieu Nacher (Université de Guyane, French Guiana), Muhayimpundu Ribakare (Rwanda Biomedical Centre, Rwanda), Nandi Siegfried (Independent Clinical Epidemiologist), Kenly Sikwese (African Community Advisory Board, Zambia), Nini Tun (Medical Action Myanmar, Myanmar), Jose E. Vidal (Instituto de Infectologia Emílio Ribas and Universidade de São Paulo, Brazil).

***Financial support.*** The development of the WHO guidelines for the management of advanced HIV disease and this supplement was supported by several sources of funding to the HIV Department, mainly funding from the US President’s Emergency Plan for AIDS Relief through the US CDC cooperative agreement and the Bill & Melinda Gates Foundation. G. M. is supported by the Wellcome Trust (grant number 098316) and the South African Research Chairs Initiative of the Department of Science and Technology and National Research Foundation of South Africa (grant number 64787). This supplement was supported by funds from the Bill & Melinda Gates Foundation.

***Supplement sponsorship.*** This article appears as part of the supplement “Advanced HIV Disease,” sponsored by the World Health Organization.

***Potential conflicts of interest.*** All authors: No reported conflicts of interest. All other authors report no potential conflicts of interest. All authors have submitted the ICMJE Form for Disclosure of Potential Conflicts of Interest. Conflicts that the editors consider relevant to the content of the manuscript have been disclosed.
